# Supplementary figures and images for: How IGF-1 activates its receptor
Source: eLife. 2014 Sep 25;3:e03772. doi: 10.7554/eLife.03772 (PMC4381924; doi:10.7554/eLife.03772)

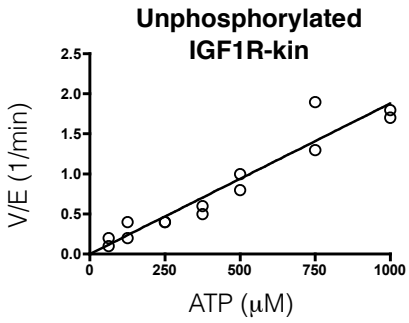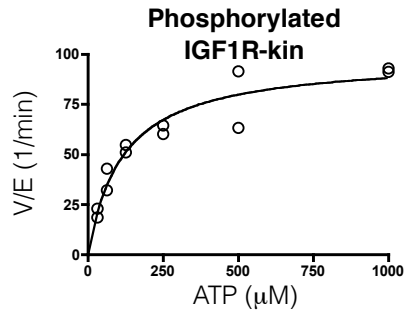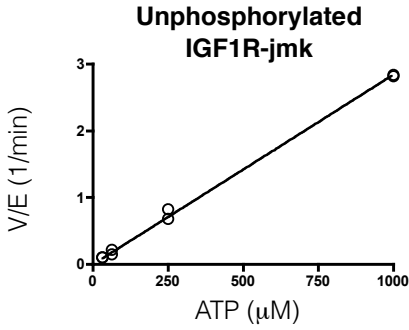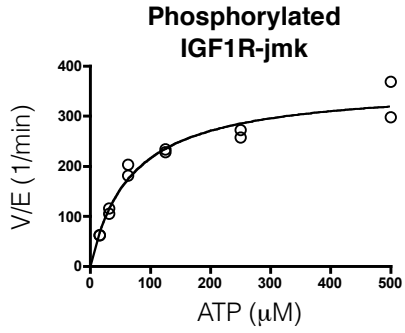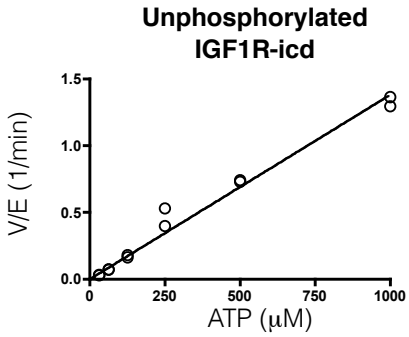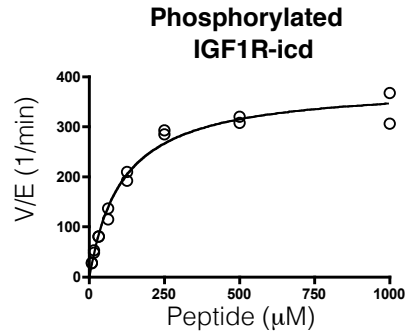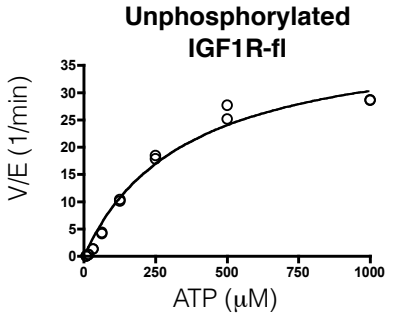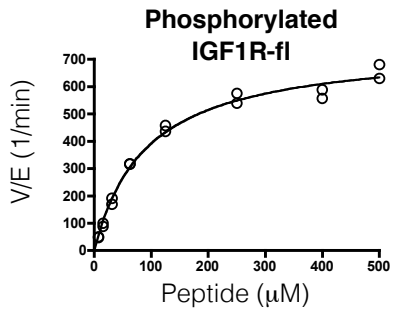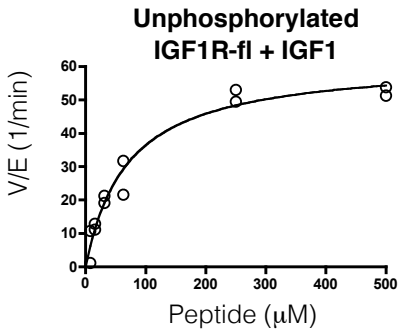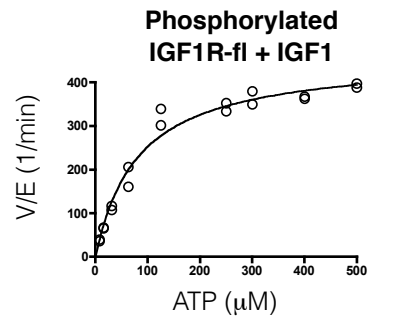

Supplement: Table 1—source data 1. — Each data point was performed in duplicate and is shown separately. DOI: http://dx.doi.org/10.7554/eLife.03772.022 [file elife03772s001.pdf]

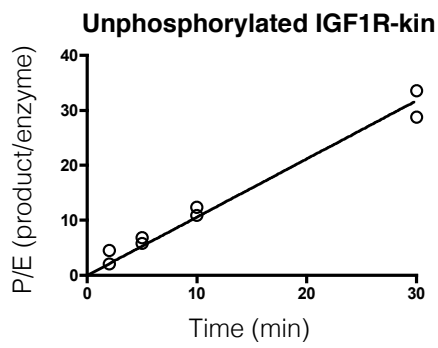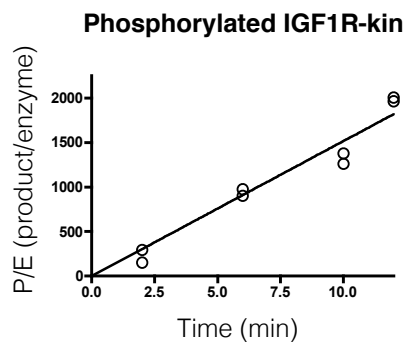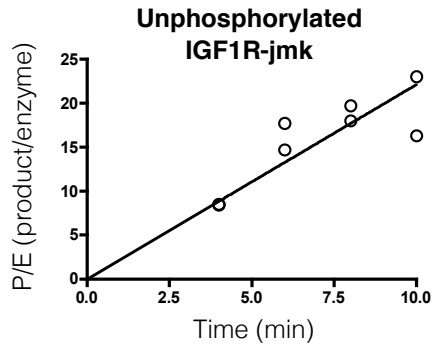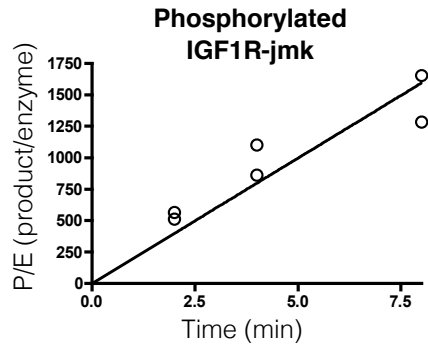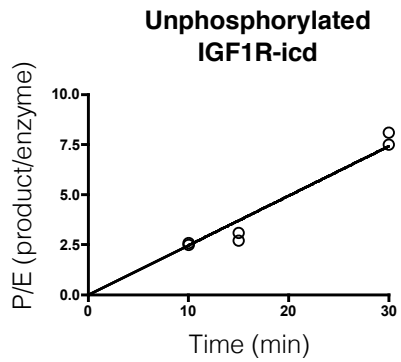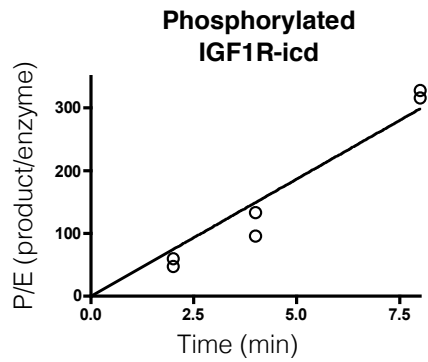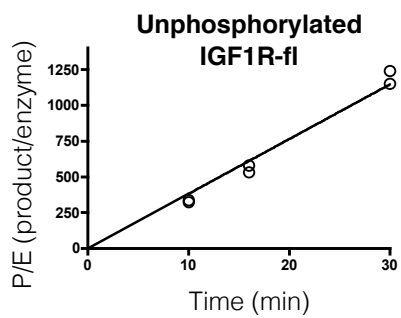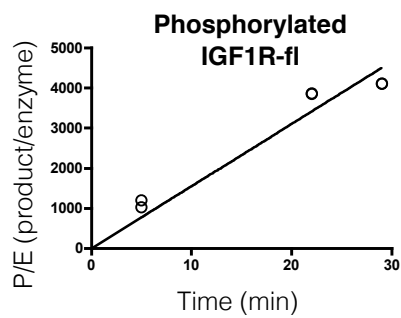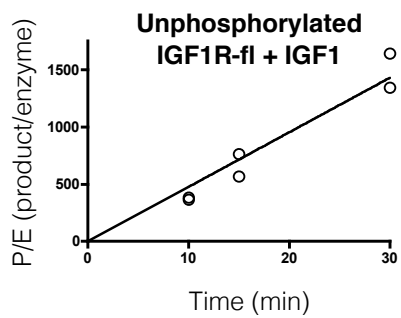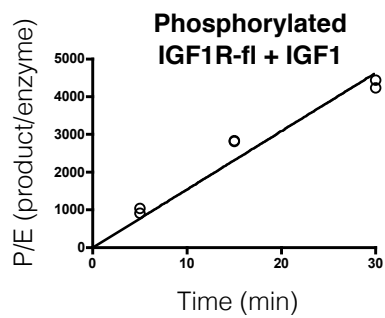

Supplement: Table 1—source data 2. — Product/Enzyme plotted vs time (minutes) for each IGF1R protein investigated. DOI: http://dx.doi.org/10.7554/eLife.03772.023 [file elife03772s002.pdf]

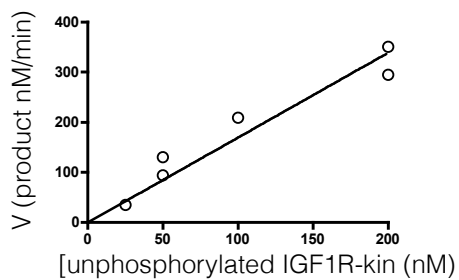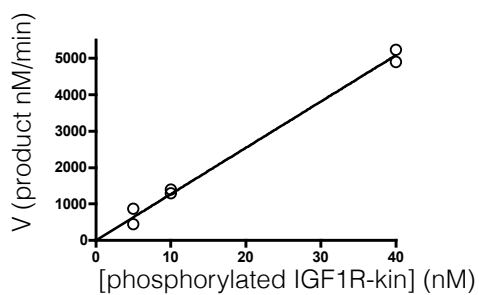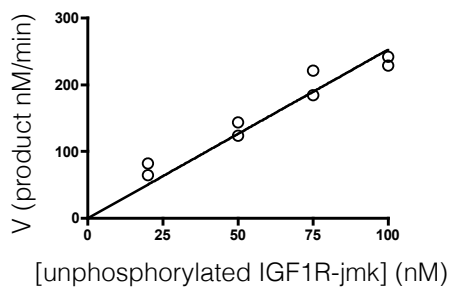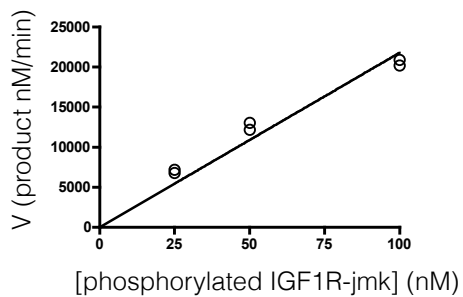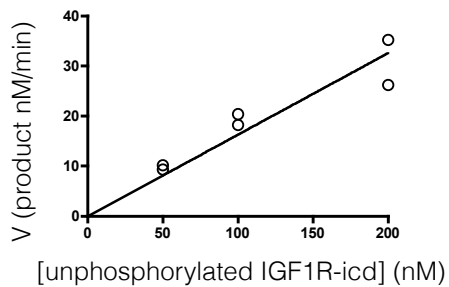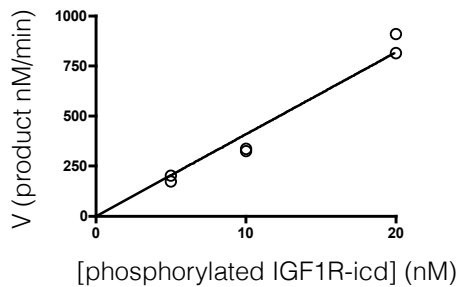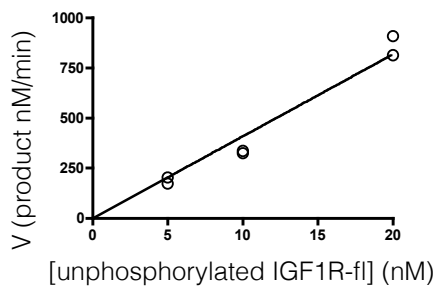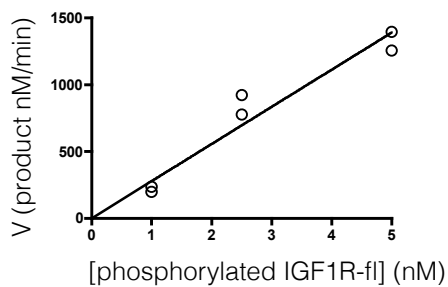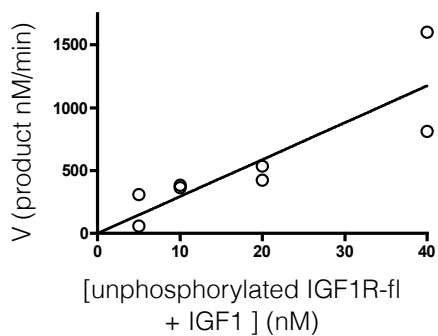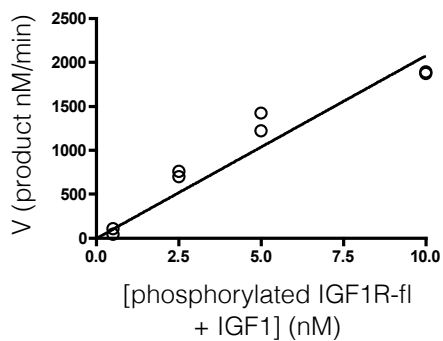

Supplement: Table 1—source data 3. — Velocity (nM of product/min) plotted vs enzyme concentration (nM) for each IGF1R protein investigated. DOI: http://dx.doi.org/10.7554/eLife.03772.024 [file elife03772s003.pdf]
